# Short-term actions of arsenic trioxide on metabolic fluxes in the isolated perfused rat liver

**DOI:** 10.1007/s00210-026-05458-8

**Published:** 2026-05-23

**Authors:** Nairana Mithieli de Queiroz Eskuarek Melo, Rosane Marina Peralta, Lívia Bracht, Adelar Bracht

**Affiliations:** Department of Biochemistry, State University of Maringá, 87020900 Maringá, PR Brazil

**Keywords:** Arsenic trioxide toxicity, Gluconeogenesis, Glycolysis, Fructolysis, Pyruvate carboxylation

## Abstract

Arsenic trioxide has recently been approved for the treatment of acute promyelocytic leukemia, but it is also a highly toxic compound. The purpose of the present work was to obtain a general view about the acute effects of arsenic trioxide on liver metabolism using the isolated perfused rat liver, a system that preserves microcirculation and cell polarity. Hepatic lactate and alanine gluconeogenesis were inhibited by arsenic trioxide with IC_50_ values of 21.7 and 21.4 µM, respectively. Oxygen uptake was inhibited only at concentrations above the IC_50_ value for gluconeogenesis inhibition. In addition to the carbon fluxes derived from alanine metabolism, arsenic trioxide also inhibited nitrogen detoxification (urea production) with an IC_50_ of 47.9 µM. Glycolysis from endogenous glycogen was stimulated at concentrations of up to 25 µM. Fructose metabolism was also affected: transformation into glucose was inhibited and fructolysis was stimulated. Glycerol metabolism was not modified. The ATP levels were not significantly diminished, but arsenic trioxide inhibited pyruvate carboxylase and phosphoenolpyruvate carboxykinase, phenomena that seem to be the main cause for gluconeogenesis inhibition. The acute effects of arsenic trioxide described herein are likely to contribute significantly to the general toxicity of the compound especially when combined with the reported long-term induction of exacerbated reactive oxygen species (ROS) production. When using the compound as a therapeutic agent, extreme care must be taken to avoid even mild overdosing as harmful and therapeutic levels for acute promyelocytic leukemia treatment are in fact relatively close to each other.

## Introduction

**Fig. 1:**
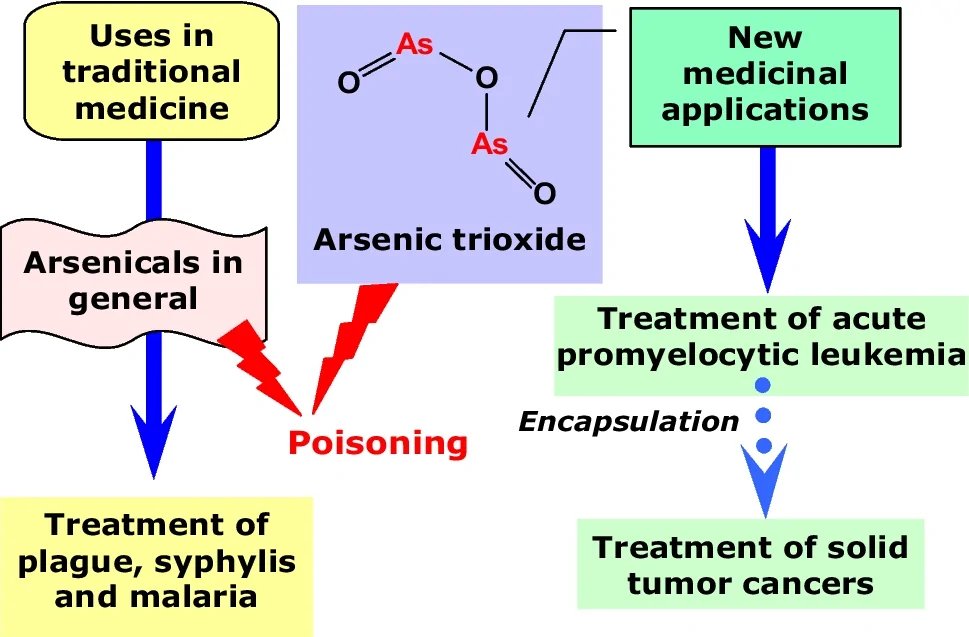
Arsenic trioxide past and present uses according to literature reports (Hoonjan et al. 2018; Yan et al. 2023)

Arsenic trioxide (As_2_O_3_; Fig. 1), as well as several other arsenic compounds, has been traditionally used as a poison, but also against a series of ailments such as ulcers (syphilis), plague, and malaria (Emadi and Gore 2010; Frith 2013; Jiang et al. 2023; Hoonjan et al. 2018). When administered to humans, depending on the dose, it can be extremely toxic and fatal. The compound is initially hydrolyzed into its trivalent form, iAs (III), which is subsequently converted into methylated trivalent metabolites such as monomethylarsonite or dimethylarsonite in addition to their pentavalent forms (Hirano et al. 2004).

One of the main toxic effects attributed to As_2_O_3_ is the induction of an exacerbated production of reactive oxygen species (ROS), especially in the liver (Ling et al. 2017). This action is prominent in the liver but has also been demonstrated to occur in several cell types at concentrations between 1 and 300 µM (Shi et al. 2004). The mechanisms are still not entirely clear, but the available evidence points out that mitochondria are the main source of the reactive oxygen species produced in excess. Excessive ROS may compromise the mitochondrial integrity, with losses in the electrochemical gradient across the membranes, activation of apoptotic pathways, and the release of cytochrome c to the cytosol (Ling et al. 2017). Reactive oxygen species contribute to depolarization of the mitochondrial membrane, a phenomenon that, in turn, activates apoptotic pathways dependent on caspases (Yan et al. 2023). Furthermore, it is believed that ROS production plays a central role in the mechanisms related to carcinogenesis induced by arsenic trioxide (Huang et al. 2004). Among the mitochondrial components involved, ubiquinone (coenzyme Q_10_) seems to play an important role in the formation of ROS. Consistently with the hypothesis, it was shown that rotenone, which inhibits the transfer of electrons in complex I to the ubiquinone, was able to suppress ROS production induced by arsenic trioxide to a significant extent (Corsini et al. 1999).

Surprisingly, and despite being toxic and even carcinogenic at high doses, arsenic trioxide gained a new role as a therapeutic agent. More specifically, it was discovered in the 1970 s that arsenic trioxide exerts a therapeutic effect on acute promyelocytic leukemia (Hoonjan et al. 2018; Jiang et al. 2023), as illustrated by Fig. 1. Several studies have demonstrated its efficiency in monotherapy, with elevated complete remission rates in both recently diagnosed patients and in patients resistant to conventional treatments (Soignet et al. 1998; Shigeno et al. 2005). In the early 2000 s, arsenic trioxide was officially approved by the Food and Drug Administration (FDA) for the treatment of promyelocytic leukemia and is presently commercialized in its injectable formulation under the name Phenasen® or Trisenox® (Australian Government 2014; Jiang et al. 2023). In addition to its use against promyelocytic leukemia, arsenic trioxide has been investigated for its therapeutic potential in other types of cancer, for example, breast cancer (Shen et al. 1997; Kumana et al. 2002), gliomas, and hepatocellular carcinomas (Emadi and Gore 2010).

In spite of its utility in cancer treatment, arsenic trioxide evidently suffers many limitations due to its toxicity and side effects. Hepatotoxicity is one of the adverse actions more frequently reported (between 25 and 45%) during therapy with arsenic trioxide (Hao et al. 2013; Wang et al. 2020). Typically, there is an elevation in the serum levels of alanine aminotransferase (ALT) and aspartate aminotransferase (AST) during the first 5 to 10 days after starting arsenic trioxide administration (Wang et al. 2020). In general, toxicity in consequence of chemotherapy with arsenic trioxide is more pronounced in tissues rich in mitochondria, an observation that has been interpreted as a consequence of the enhanced mitochondrial ROS production induced by the compound (Ling et al. 2017). Besides, enhanced ROS production can also induce structural modifications in DNA, such as mutations in base pairs, translocations, and deletions (Shi et al. 2004).

With respect to metabolism, there is a study conducted with isolated renal tubules and isolated hepatocytes in which inhibition of gluconeogenesis by arsenic trioxide was detected (Szinicz and Forth 1988). The authors concluded that the mechanism of this effect may have multiple causes, especially because the degree of inhibition varied substantially with the gluconeogenic precursor. The study was conducted using closed and relatively long incubations, a procedure in which it is difficult to distinguish between short-term and long-term effects. The first ones are generally consequences of the action of the parent substance whereas the latter may result from the accumulation of metabolites, which in the case of arsenic trioxide includes even reactive oxygen species. With this fact in mind, we decided to conduct a more detailed study on the acute effects of arsenic trioxide on several metabolic fluxes in the liver using the isolated perfused rat liver as the experimental model (Scholz and Bücher 1965; Kelmer-Bracht et al. 1984). This experimental system allows to measure short-term modifications in steady-state rates under conditions in which both cell polarity and the microcirculation are preserved. In addition to gluconeogenesis, several other metabolic routes, such as glycolysis, fructolysis, glycogenolysis, and ureagenesis, were measured under the influence of various arsenic trioxide concentrations. This more ample approach will allow to get a more complete vision of the acute metabolic effects of arsenic trioxide and will also provide a comparison basis with the effects of sodium arsenite and sodium arsenate, which were investigated in detail in a recent study (Melo et al. 2024).

## Materials and methods

### Materials

Enzymes, coenzymes, and arsenic trioxide were obtained from Sigma-Aldrich Co (St. Louis, USA). Chemical grade compounds were 98–99.8% pure.

### Animals

Male albino Wistar rats (250 ± 30 g) were used. Animals were maintained on a regulated light–dark cycle, under constant temperature (22 ± 3 °C) and were fed ad libitum with a standard laboratory diet (Nuvilab®, Colombo, Brazil). Anesthesia was brought about by intraperitoneal administration of ketamine (70 mg/kg) + xylazine (7 mg/kg) before preparing the liver for perfusion (Scholz and Bücher 1965; Kelmer-Bracht et al. 1984). The experiments were forehand approved by the Ethics Committee of Animal Experimentation of the University of Maringá on 03/16/2023 (protocol #5,386,010,223).

Experiments in which gluconeogenesis was measured were conducted with livers from rats after 18-h fasting. Hepatic glycogen levels under these conditions are minimal and glucose release reflects the gluconeogenic activity (Comar et al. 2016). When glycogen catabolism was investigated, livers from fed rats were used. The number of rats used in each experimental series is informed in the legends to the figures.

### Liver perfusion

Once-through perfusion was done (Scholz and Bücher 1965; Kelmer-Bracht et al. 1984). The perfusion liquid (Krebs/Henseleit-bicarbonate buffer, pH 7.4) contained 0.25 g/L of bovine serum albumin and was equilibrated with an oxygen + carbon dioxide atmosphere (95:5). The latter was automatically accomplished when the perfusion fluid was pumped at a constant rate through the oxygenator. The latter consisted of 15 m of thin-walled silicon rubber tubing wound around a thermostated aluminum core (37 °C). Perfusion was accomplished by conducting the oxygenated and warmed up perfusion fluid into the portal vein by means of a plastic cannula. The perfusion rate was between 30 and 32 mL/min. Outflowing of the perfusion fluid occurred via the cava vein. The substrates (L-lactate, alanine, glycerol, and fructose) and arsenic trioxide were dissolved in the perfusion liquid at the desired concentrations. Samples for quantifying glucose, lactate, and pyruvate were collected from the outflowing perfusion fluid (Bergmeyer 1974). Oxygen concentration was monitored continuously in the outflowing perfusion fluid by a polarographic assembly consisting in a Teflon-shielded platinum electrode, a polarograph, and a potentiometric recorder (Kelmer-Bracht et al. 1984). Sampling of the outflowing perfusate was initiated after stabilization of oxygen uptake, which reflects the recovery of the liver from the short period of anoxia (10 min) caused by the low perfusion flow during the period where the liver is cannulated and transferred to the perfusion device.

### Quantification of liver ATP

The hepatic contents of ADP and ATP were measured in liquid nitrogen freeze-clamped livers. The freeze-clamped livers were extracted with trichloroacetic acid. The pH of the extract was neutralized with K_2_CO_3_ and ADP, and ATP were assayed by means of high-performance liquid chromatography (HPLC) (Mito et al. 2014). The HPLC system (Shimadzu, Japan) consisted of a system controller (SCL-10AVP), two pumps (model LC10ADVP), a column oven (model CTO-10AVP), and an UV–Vis detector (model SPD-10AV). A reversed-phase C18 CLC-ODS column (5 µm, 250 × 4.6 mm i.d., Shimadzu) protected with a CLC-ODS precolumn (5 µm, 4 × 3 mm i.d., Phenomenex) was used with a gradient from reversed-phase 0.044 mol/L phosphate buffer solution, pH 6.0, to 0.044 mol/L phosphate buffer solution plus methanol (1.1), pH 7.0. In percent methanol, the gradient was the following: at 0 min, 0%; at 2.5 min, 0.5%; at 5 min, 3%; at 7 min, 5%; at 8 min, 12%; at 10 min, 15%; at 12 min, 20%; at 20 min, 28%; and at 30 min, 0%. The temperature was kept at 35 °C, and the injection volume was 20 µL with a flow rate of 0.8 mL/min. Monitoring was performed spectrophotometrically at 254 nm. Identification of the peaks of the investigated compounds was carried out by comparison of their retention times with those obtained by injecting standards under the same conditions. The concentrations of the compounds were calculated by means of the regression parameters obtained from the calibration curves. The calibration curves were constructed by separating chromatographically standard solutions of the compounds. Linear relationships were obtained between the concentrations and the areas under the elution curves.

### Cell fractionation and enzyme assays

For cell fractionation the livers were removed from previously anesthetized rats and cut into small pieces. After homogenization in an isolation medium that consisted of 0.2 M mannitol, 75 mM sucrose, 2 mM Tris–HCl (pH 7.4), 0.2 mM EGTA, and 50 mg/100 mL bovine serum albumin, the mitochondria were isolated by differential centrifugation and the microsomes were eliminated by ultracentrifugation at 105000* g* (Bracht et al. 2003; Vilela et al. 2014; Maldonado et al. 2017). Both the isolated mitochondria and the cytosolic fraction were kept at 0–4 °C until use. The cytosolic fraction is the supernatant obtained from the centrifugation at 105,000* g* (Sá-Nakanishi et al. 2020). Protein content was measured using the Folin phenol reagent according to Lowry et al. (1951), with bovine serum albumin as standard.

The activity of the phosphoenolpyruvate carboxykinase (PEPCK) in the cytosolic fraction was estimated by coupling with the malate dehydrogenase reaction (Bracht et al. 2016). The oxidation of NADH by oxaloacetate formed in the PEPCK reaction was followed spectrophotometrically at 340 nm for 2 min, and the enzyme activity was expressed as nmol NADH oxidized per minute per milligram protein.

The pyruvate carboxylase of disrupted mitochondria was measured using a medium able to generate steady-state concentrations of acetyl-CoA (Henning et al. 1966; Eler et al. 2009). Rat liver mitochondria, isolated as described above, were disrupted by successive freeze and thawing procedures using liquid nitrogen. The incubation medium contained 6 mg protein/mL of disrupted mitochondria, 10 mM sodium pyruvate, 12.5 mM MgCl_2_, 2.5 mM potassium phosphate, 0.3 M sucrose, 1 mM ethylenediamine tetraacetate, 5 mM tris(hydroxymethyl)aminomethane (Tris; pH 7.5), 0.5 mM lithium coenzyme A, 5 mM adenosine triphosphate, 1.1 mM acetyl phosphate, 6 µg/mL phosphotransacetylase, and 12 µg/mL citrate synthase. The reaction was initiated by introducing 24 mM [^14^C]NaHCO_3_ (0.50 µCi). After 10 min of incubation at 37 °C, the reaction was arrested by the addition of a 0.5 volume of 2 N perchloric acid. After expulsion of the remaining [^14^C]NaHCO_3_ (5 min), aliquots were taken for counting the acid-stable incorporated radioactivity. The incorporated radioactivity was expressed as nmol min^−1^ mg protein^−1^. The scintillation liquid for counting ^14^C was a mixture of toluene/Triton X-100® (1.5/0.5), containing 10 g/L 1,5-diphenyloxazole and 0.4 g/L 2,2-*p*-phenylene-bis(5-phenyloxazole).

### Mitochondrial respiration assay

Mitochondrial oxygen consumption was measured by polarography using a Teflon-shielded platinum electrode (Voss et al.^1961^; Bracht et al. 2003; Bonetti et al. 2025). The mitochondria were incubated in a closed oxygraph chamber in a medium (2.0 mL) containing 0.25 M mannitol, 5 mM sodium phosphate, 10 mM KCl, 0.2 mM EDTA, and 10 mM Tris–HCl (pH 7.4). Arsenic trioxide was added for final concentrations between 5 and 100 µM. The substrates succinate and glutamate were used at final concentrations of 10 mM. The oxygen consumption rates were calculated from the slopes of the recording tracings and expressed as nmol × min^−1^ × (mg protein)^−1^. The respiratory rates were measured under two different conditions: (a) before ADP addition (substrate or basal respiration); (b) just after ADP addition for a final concentration of 0.125 mM (state III respiration). The ADP/O ratio (ADP added divided by the amount of oxygen consumed during the ADP-stimulated respiration) was calculated from these data (Voss et al. 1961; Bonetti et al. 2025).

### Data handling

The results were presented as means ± standard errors of the mean. The concentration dependences of the effects of arsenic trioxide on the metabolic fluxes were submitted to variance analysis (ANOVA) followed by post hoc testing according to Student–Newman–Keuls (*p* ≤ 0.05), using the Statistica® software from StatSoft (Tulsa, USA). Differences between two means were assessed by Student’s *t* test. IC_50_ values (concentrations for half-maximal inhibition) were computed by numerical interpolation using Stineman’s interpolation formula.

## Results

### Glycogen catabolism

**Fig. 2:**
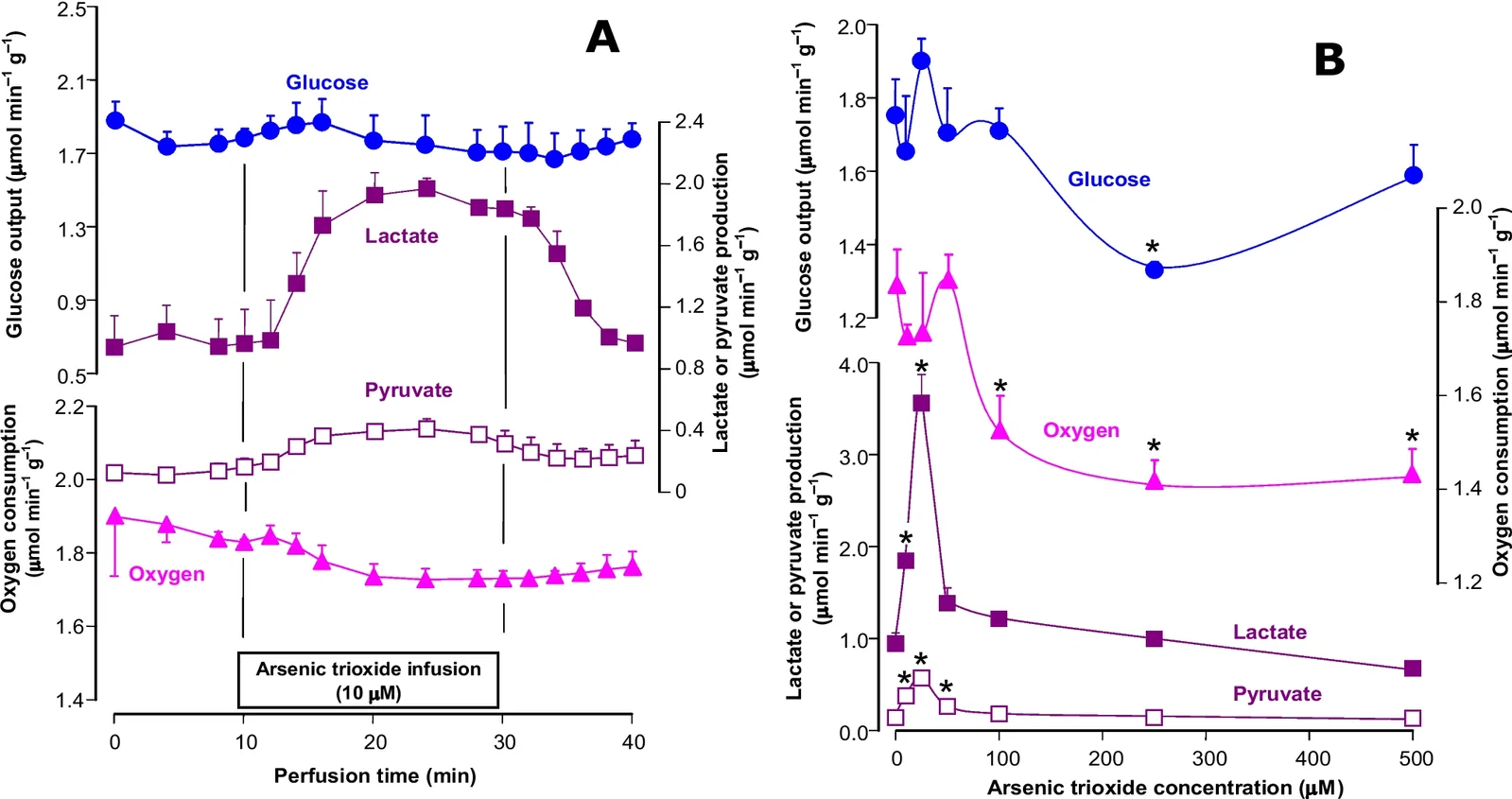
The action of arsenic trioxide on glycogen catabolism and related parameters in the isolated perfused rat liver. Livers of fed rats were perfused with substrate-free Krebs/Henseleit-bicarbonate buffer, as described in “Materials and methods.” Panel **A** shows the time course of the effects of 10 µM arsenic trioxide. Times of lactate and arsenic trioxide infusion are indicated. Panel **B** presents the concentration dependences of the effects on the arsenic trioxide concentrations in the range between 10 and 500 µM. Experimental points are means ± mean standard errors of 3 liver perfusion experiments

Livers from fed rats present high contents of glycogen, a situation that allows to measure various parameters related to the catabolism of this polysaccharide. When livers from fed rats are perfused with substrate-free medium, respiration occurs mainly at the expense of their high endogenous content of fatty acids (Scholz and Bücher 1965; Kelmer-Bracht et al. 1984; Comar et al. 2016). They also release glucose (glycogenolysis) and lactate plus pyruvate (glycolysis) at the expense of their endogenous glycogen stores. Figure 2 illustrates the response of livers to arsenic trioxide under these conditions. Panel A shows the time course of the experiments that were done with 10 µM arsenic trioxide and also illustrates the experimental protocol that was followed with all other concentrations of the former arsenical. Sampling of the outflowing perfusate was started after oxygen uptake stabilization and was taken as zero perfusion time. Glucose release (from glycogenolysis), lactate and pyruvate production (glycolysis), and oxygen uptake had attained steady-state rates previous to the arsenic trioxide infusion which occurred at 10-min perfusion time. At the concentration of 10 µM, arsenic trioxide did not modify glucose output, nor did it affect oxygen uptake. Lactate and pyruvate productions, however, were both increased. These increases were stable during the whole infusion period. Interruption of the infusion at 30-min perfusion time was followed by a return of both parameters to the levels observed before the infusion was started. This means that the effects of arsenic trioxide are reversible. Experiments like those illustrated by panel A in Fig. 2 were repeated over an ample concentration range. Panel B summarizes the results. Lactate and pyruvate production stimulation reached a maximum at the concentration of 20 µM. For lactate production, the stimulation was around 3.5-fold. There was still some stimulation at the concentration of 50 µM, but for all concentrations higher than the latter stimulation did no longer occur. Stimulations of lactate and pyruvate productions were always reversible, i.e., cessation of the infusion restored the glycolytic flux to the control levels. Quite different effects were found for glucose output and oxygen consumption. No significant modifications were seen with concentrations smaller than 100 µM. Oxygen uptake, however, was clearly inhibited within the concentration range between 100 and 500 µM. Glucose output also tended to be diminished at high concentrations, although this effect was not as well defined as the action on oxygen uptake.

### Lactate gluconeogenesis

**Fig. 3:**
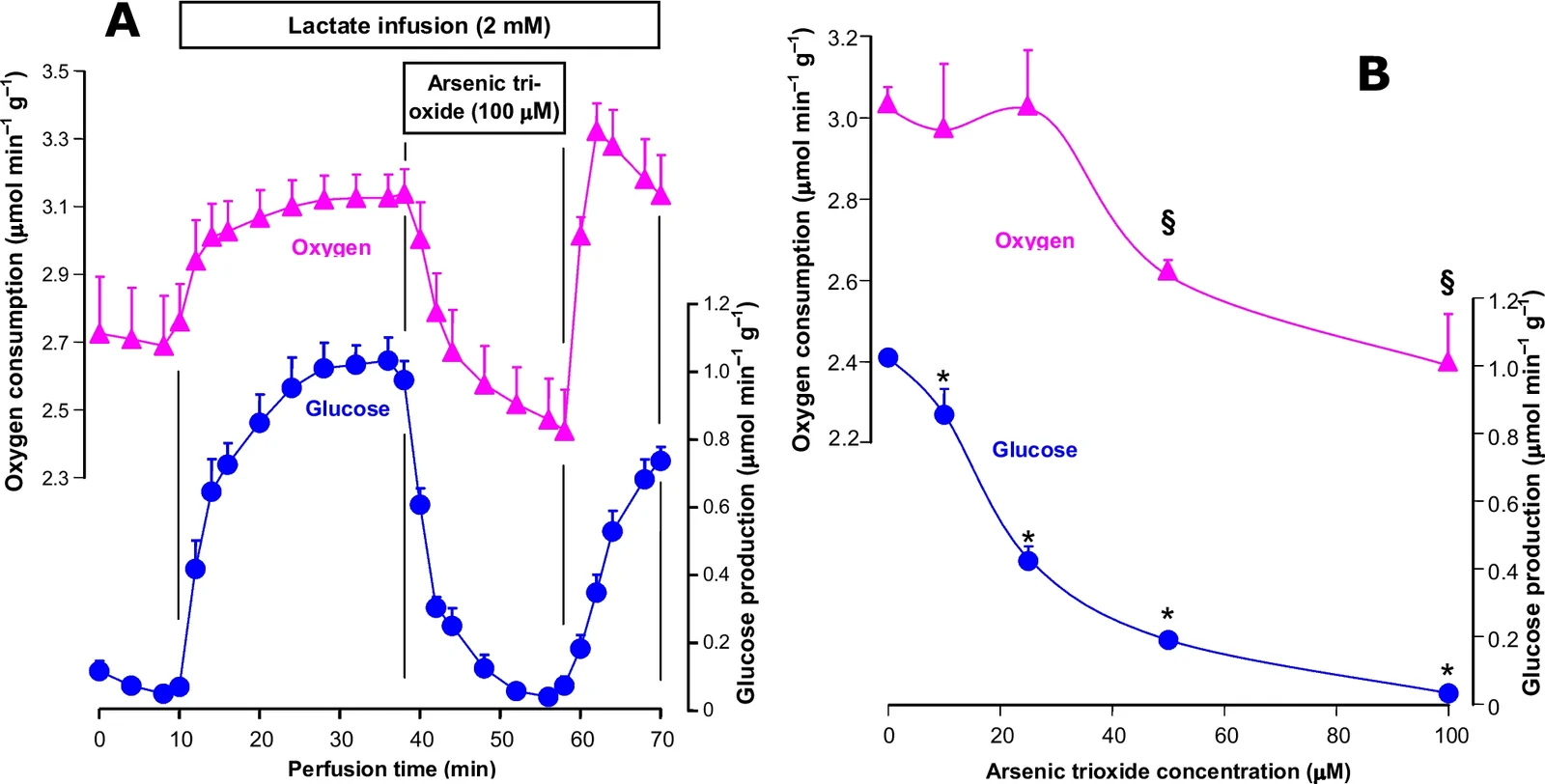
Actions of arsenic trioxide on lactate gluconeogenesis and the associated oxygen consumption in the isolated perfused rat liver. Panel **A** shows the time course of the effects of arsenic trioxide on oxygen consumption and glucose output at the concentration of 100 µM. Times of lactate and arsenic trioxide infusion are indicated. Panel **B** presents the concentration dependences of the effects on the portal arsenic trioxide concentrations in the range between 10 and 100 µM. Livers from 18-h fasted rats were used. The experimental points are means of three independent liver perfusion perfusion experiments for each concentration. Asterisks (*) and section signs (§) above the experimental points in panel **B** indicate statistically significant differences in comparison with the control conditions (zero arsenic trioxide in the inflowing perfusate)

The transformation of lactate into glucose (gluconeogenesis) can be measured when the hepatic glycogen stores are negligibly small, a phenomenon that occurs in the liver of rats after a 18-h fasting (Comar et al. 2016). This pathway is actually the opposite of the transformation of glucose derived from glycogen into lactate. To see how arsenic trioxide affects lactate gluconeogenesis, the substrate was infused in livers from 18-h fasted rats at the concentration of 2 mM. After 30 min, arsenic trioxide was infused cumulatively at various concentrations. The results of this series of experiments are shown in Fig. 3. Glucose output was minimal before starting the infusion of lactate and oxygen uptake was at a steady-state level. The sudden introduction of lactate caused progressive increases in both glucose production and oxygen uptake until reaching new steady-state levels, as shown in panel A of Fig. 3. The introduction of 100 µM arsenic trioxide caused a progressive decrease in both oxygen uptake and glucose production. Inhibition of glucose production was actually almost complete at 58-min perfusion time and oxygen uptake fell to levels below the basal ones, i.e., before the initiation of lactate infusion. Upon the interruption of arsenic trioxide infusion, both oxygen uptake and glucose production recovered, meaning reversibility of the inhibitory effects. In order to get a more complete picture of the effects of arsenic trioxide on gluconeogenesis, the experiment shown in panel A was repeated with lower arsenic trioxide concentrations. Concentrations higher than 100 µM were not used because the latter already caused almost 100% inhibition of glucose production. The concentration dependences are shown in panel B of Fig. 3. Inhibition of glucose production was already significant at the concentration of 10 µM. An IC_50_ value of 21.7 µM was computed by numerical interpolation. Oxygen uptake, on the other hand, was not inhibited by concentrations up to 20 µM. Concentrations of 50 and 100 µM, however, were inhibitory.

### Alanine metabolism

**Fig. 4:**
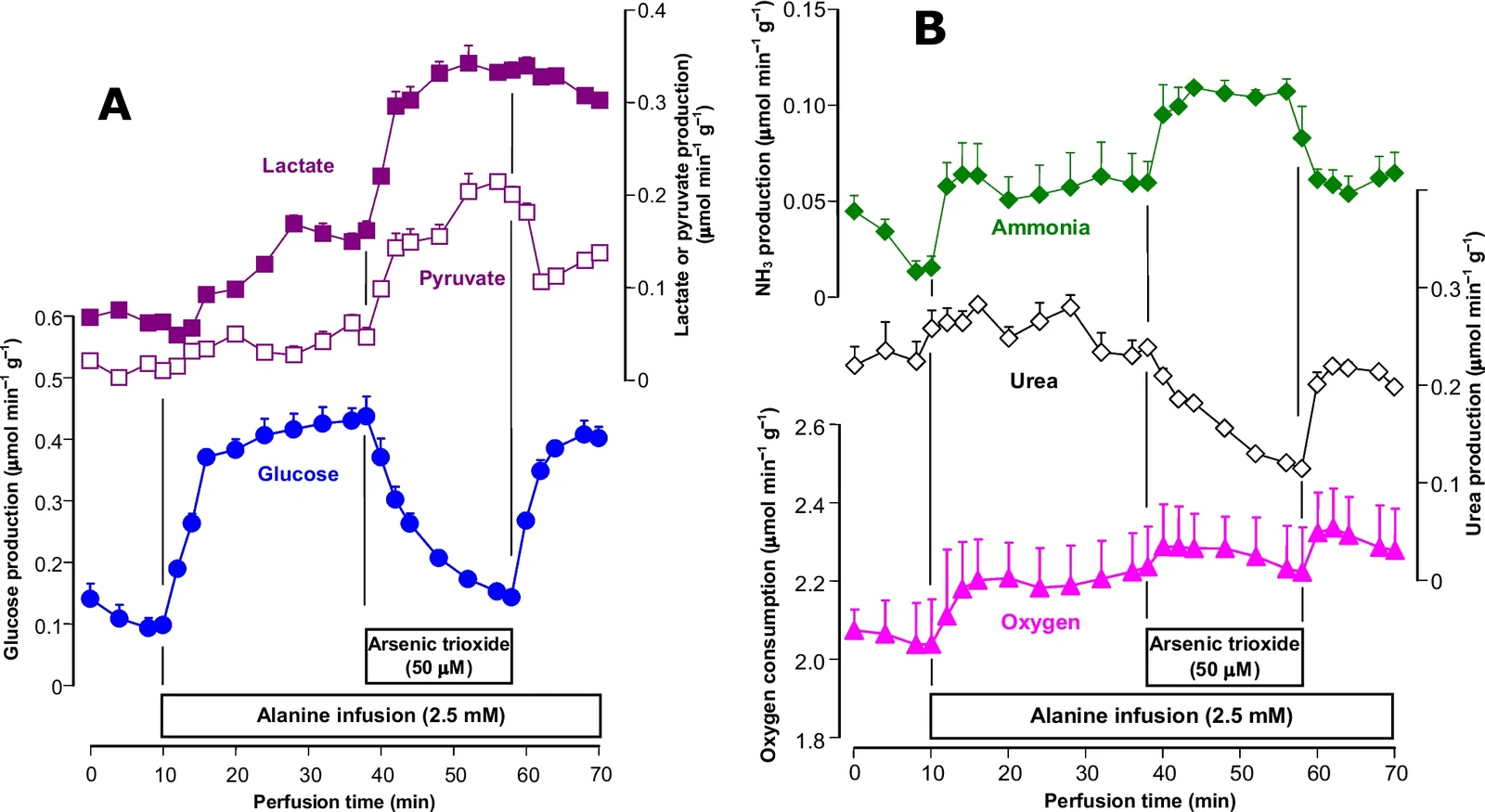
Time courses of the effects of 50 µM arsenic trioxide on parameters derived from alanine metabolism in the isolated perfused rat liver. Livers from 18-h fasted rats were perfused as described in “Materials and methods.” Times of alanine and arsenic trioxide infusion are indicated. The experimental points are means ± mean standard errors of three independent liver perfusion experiments for each concentration. The parameters represented in each panel are indicated. The experimental points are means of three independent liver perfusion perfusion experiments

**Fig. 5:**
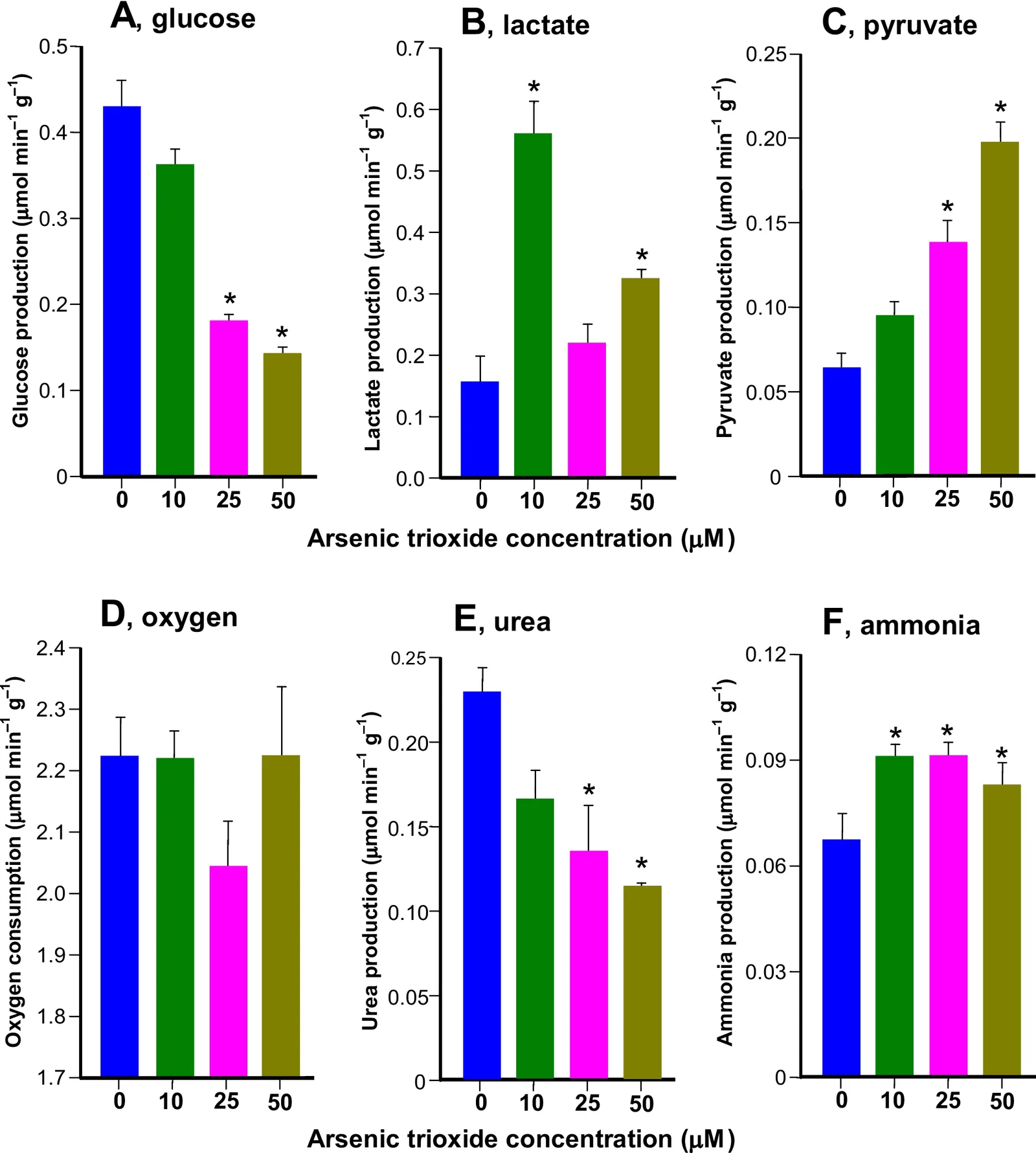
Comparison of the effects of three concentrations of arsenic trioxide (15, 25, and 50 µM) on parameters related to alanine metabolism in the perfused rat liver. The data were obtained from experiments conducted according to the protocol illustrated by Fig. 4. Asterisks indicate statistically significant differences in comparison with the control conditions (zero arsenic trioxide in the inflowing perfusate)

Measurement of alanine transformation yields useful data because the metabolism of this compound generates both carbon and nitrogen fluxes. How arsenic trioxide affects alanine metabolism can be inferred from Figs. 4 and 5. Figure 4 shows representative time courses of the changes; and Fig. 5 the concentration dependences of the effects. Panel A of Fig. 4 shows that the introduction of alanine produced, as expected, a large increase in glucose production. There was also a clear increase in lactate production and a very modest increase in pyruvate production. The subsequent infusion of 50 µM arsenic trioxide, at 38-min perfusion time, progressively decreased glucose production to minimal values; upon withdrawal of the arsenical, at 58-min perfusion time, the production of glucose was progressively restored. Lactate and pyruvate production, on the other hand, were increased by the infusion of arsenic trioxide. Upon withdrawal of the arsenical, the stimulated pyruvate production diminished rapidly; diminution of the stimulated lactate production, however, was still in progress when the perfusion was stopped. With respect to the nitrogen fluxes, which are shown in panel B of Fig. 4, arsenic trioxide stimulated ammonia production and decreased urea production, parameters that had been previously increased by alanine. These effects were reversible. Oxygen uptake was not inhibited by arsenic trioxide, there was actually a tendency toward stimulation.

Figure 5 summarizes the effects of various concentrations of arsenic trioxide. Glucose production inhibition was concentration-dependent, with an IC_50_ of 21.4 µM. Pyruvate production stimulation also presented a clear concentration dependence; interpolation revealed that doubling of this parameter relative to the control can be expected at the concentration of 20.4 µM. Inhibition of urea production was less sensitive to arsenic trioxide than glucose production, with an IC_50_ of 47.9 µM. Lactate production stimulation presented a peak at the concentration of 10 µM and stimulation of ammonia production was relatively modest without a defined concentration dependence. No significant inhibition of oxygen uptake was found for concentrations of up to 50 µM arsenic trioxide.

### Fructose metabolism

**Fig. 6:**
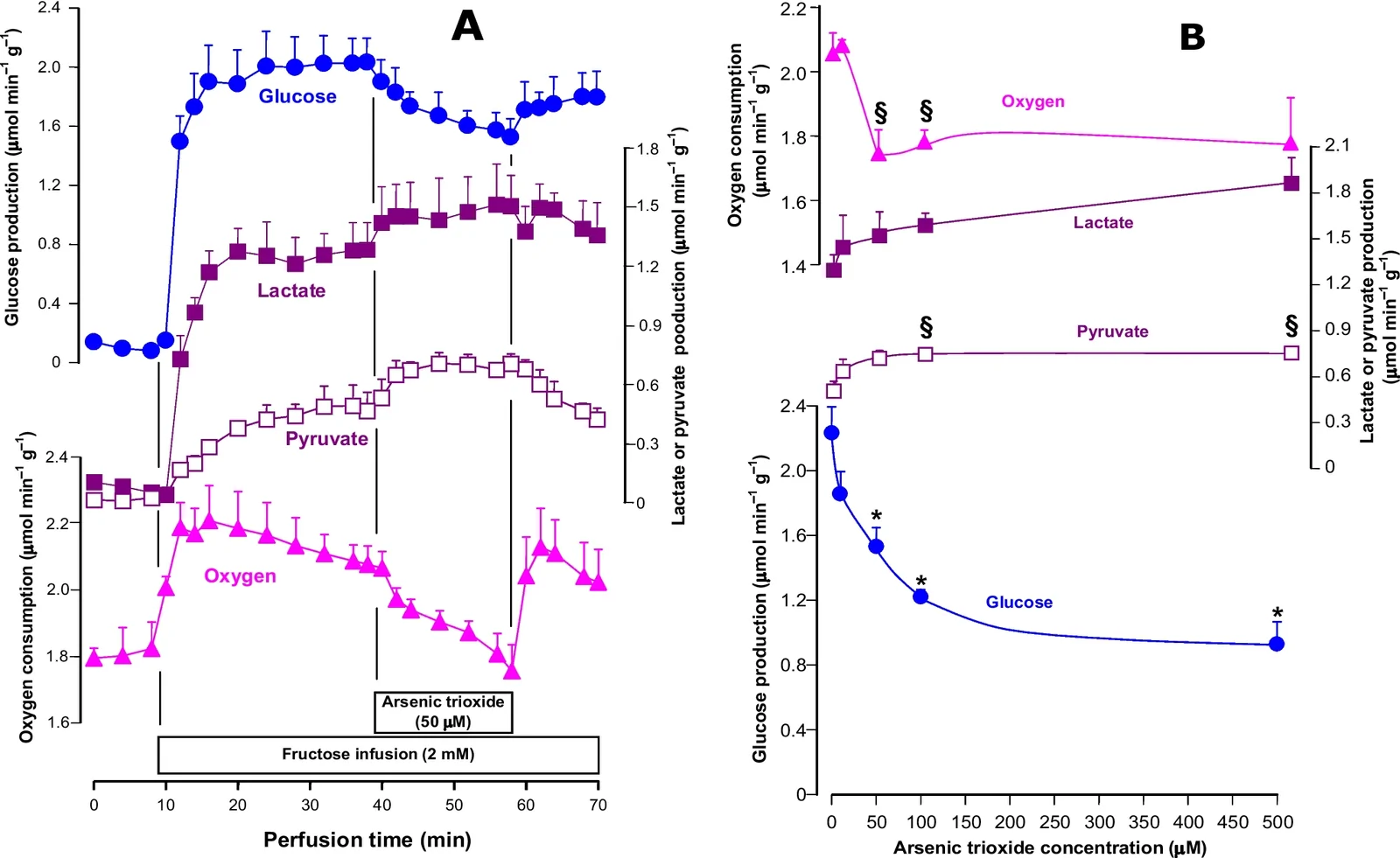
Actions of arsenic trioxide on fructose metabolism in the isolated perfused rat liver. Panel **A** shows the time course of the effects of 50 µM arsenic trioxide on the various parameters that were monitored, as indicated. Times of fructose and arsenic trioxide infusion are indicated. Panel **B** presents the concentration dependences of the effects on the portal arsenic trioxide concentrations in the range between 10 and 500 µM. Livers from 18-h fasted rats were used. The experimental points are means of three independent liver perfusion experiments for each concentration. Asterisks (*) and section signs (§) above the experimental points in panel **B** indicate statistically significant differences in comparison with the control conditions (zero arsenic trioxide in the inflowing perfusate)

Fructose metabolism in the liver is characterized by both anabolic (glucose production) and catabolic (fructolysis) pathways. In order to see if arsenic trioxide can influence simultaneously pathways that consume and produce high-energy intermediates (ATP), experiments were done employing a wide range of concentrations. The results are shown in Fig. 6; panel A illustrates the time courses of the effects of 50 µM arsenic trioxide and panel B the concentration dependences of the effects. The transformation of fructose in the liver occurs at relatively high rates, as indicated by the substantial increases in glucose, lactate, and pyruvate productions that occurred when the substrate was infused (panel A). Oxygen uptake was also increased. The introduction of 50 µM arsenic trioxide decreased glucose production and caused modest increases in lactate and pyruvate production. Oxygen uptake was also decreased. Reversibility, or at least partial reversibility, was the general rule. The concentration dependences in panel B reveal that the actions of arsenic trioxide on the metabolism of fructose seem to be much less pronounced than those on lactate and alanine metabolism. Inhibition of glucose production, nonetheless, showed a well-defined concentration dependence with an IC_50_ of 128.8 µM. Inhibition was clearly incomplete, however, as even concentrations as high as 500 µM did not produce an inhibition that was substantially higher than 50%. The modest stimulations in pyruvate production were already saturated at the concentration of 100 µM and the stimulation of lactate production did not prove to be statistically relevant. Oxygen uptake was inhibited, but showed substantial dispersion, especially at the highest concentration.

### Glycerol metabolism

**Fig. 7:**
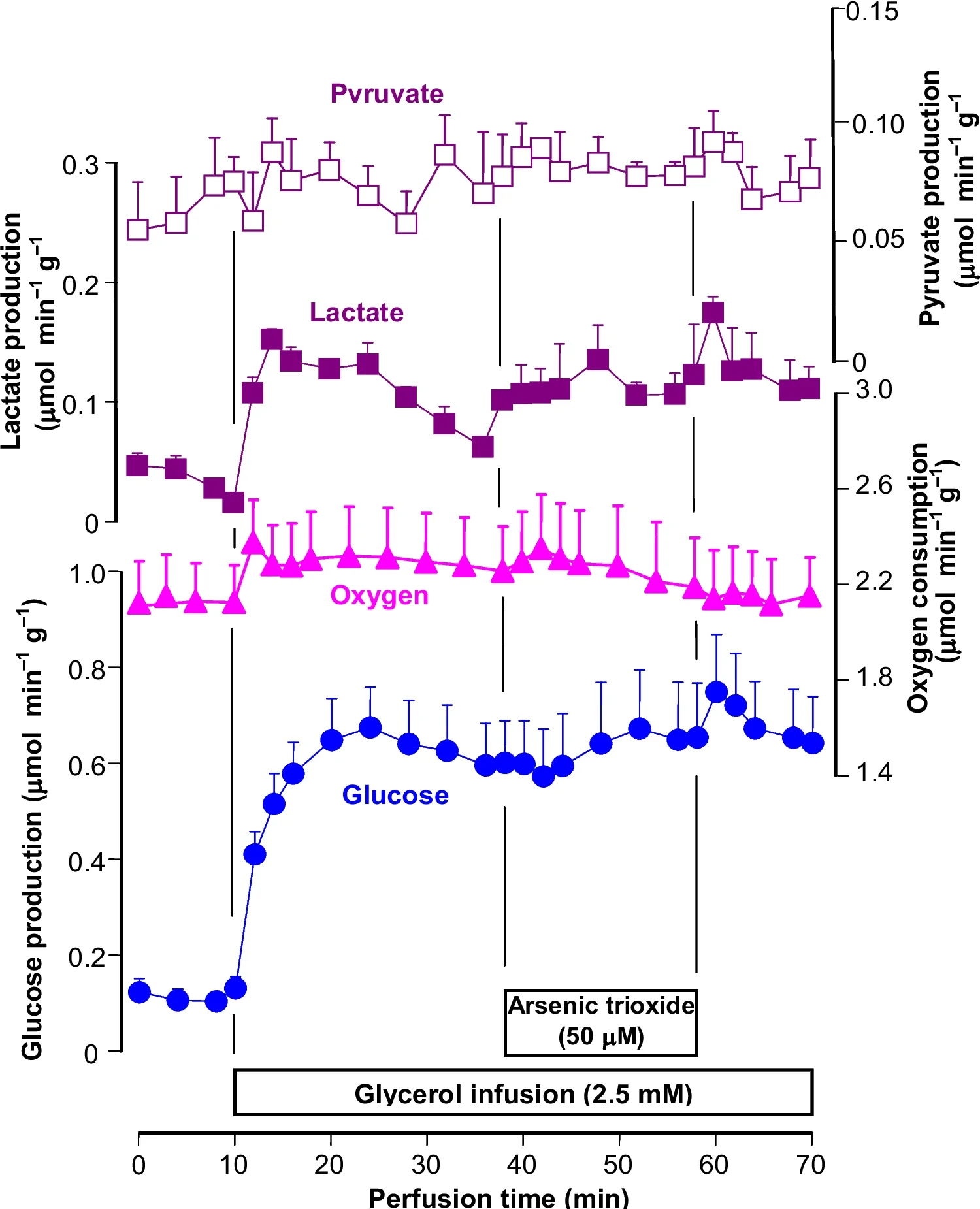
Time course of the actions of 50 µM arsenic trioxide on glycerol gluconeogenesis and associated parameters in the isolated perfused rat liver. Times of glycerol and arsenic trioxide infusion are indicated. Livers from 18-h fasted rats were used. The experimental points are means of three independent liver perfusion experiments for each concentration

The metabolism of glycerol in the liver is similar to that of fructose in that this substrate also generates anabolic (glucose synthesis) and catabolic (production of lactate and pyruvate) fluxes. The possibility that arsenic trioxide might influence glycerol metabolism was also explored and the results are shown in Fig. 7 using solely the concentration of 50 µM, which had a pronounced effect on alanine and lactate metabolism. The most important metabolic fluxes generated by glycerol are glucose and lactate production. The stimulation of lactate production by glycerol, however, showed a strong tendency of not reaching steady state. The introduction of 50 µM arsenic trioxide at 38-min perfusion time, on the other hand, did not produce significant modifications with the possible exception of lactate production which tended to be stimulated.

### Hepatic ADP and ATP levels

Oxygen uptake was strongly inhibited when the liver was producing glucose from exogenously infused lactate (see Fig. 3). This inhibition may or may not be accompanied by modifications in the ATP content of the hepatocytes. The experimental condition that was chosen to verify this hypothesis was perfusion time 58 min under the conditions given by Fig. 3A, i.e., simultaneous infusion of 2 mM lactate and 100 µM arsenic trioxide. In the control experiments, no arsenic trioxide was infused. The results are displayed in Table 1. Arsenic trioxide tended to decrease the ATP content, but statistical significance at the 0.05% level was lacking. The ADP content, however, was clearly diminished by 41.1%.

**Table 1:** Hepatic ATP and ADP contents and the effects of 50 µM arsenic trioxide. Tissue extraction, sample preparation and the chromatographic procedures are described in the Materials and Methods section. The *p* values refer to Student’s t test

Compound	Control	Arsenic trioxide (50 µM)	*p*
ATP (µmol g^−^^1^)	1.030 ± 0.120	0.813 ± 0.036	0.158
ADP (µmol g^−^^1^)	1.213 ± 0.092	0.714 ± 0.042	0.008

### Mitochondrial respiration

**Fig. 8:**
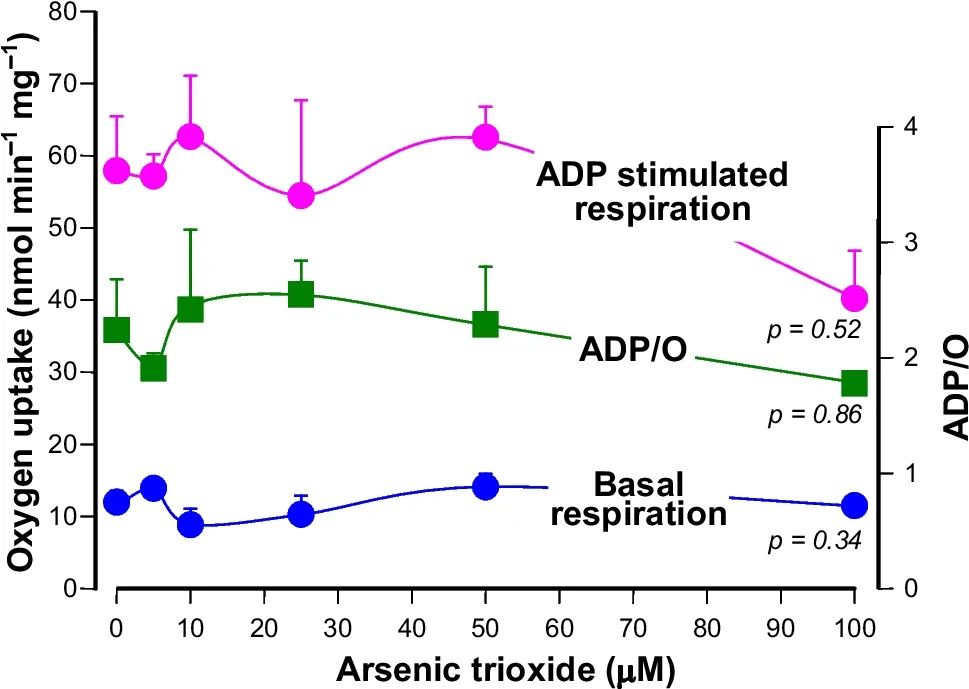
Actions of arsenic trioxide on the respiratory activity of isolated rat liver mitochondria driven by succinate (10 mM). The ADP concentration in the ADP-stimulated respiration was equal to 125 µM. The experimental details and definition of the rates and respiratory control ratio are given in “Materials and methods.” Bars are means and mean standard errors of 3 mitochondrial preparations. The *p* values refer to monovariate variance analysis for each parameter

Oxygen uptake inhibition by arsenic trioxide is a phenomenon that accompanies lactate gluconeogenesis inhibition, but not alanine gluconeogenesis inhibition or glycolysis stimulation. This suggests that the phenomenon is not a consequence of a general and direct interaction of arsenic trioxide with the mitochondrial respiratory chain. To test this hypothesis, the respiratory activity of isolated mitochondria was measured polarographically, and the results are shown in Fig. 8. Arsenic trioxide did not significantly inhibit oxygen uptake of isolated mitochondria either in the absence (basal respiration) or presence of ADP in the concentration range of up to 100 µM. Nor did it affect the ADP/O ratio, i.e., the efficiency of energy transduction.

### Effects on enzymes of the gluconeogenic pathway

The action of arsenic trioxide on the activities of phosphoenolpyruvate carboxykinase and pyruvate carboxylase was measured because these enzymes catalyze key regulatory steps of the gluconeogenic pathway. The results are shown in Table 2. Arsenic trioxide, at the concentration of 50 µM, inhibited the phosphoenolpyruvate carboxykinase activity by 43.1%. The pyruvate carboxylase, on the other hand, was extremely sensitive to arsenic trioxide; more specifically, 99.6% inhibition was found at the concentration of 50 µM. The most important phosphatase of the gluconeogenic pathway, i.e., fructose 1,6-bisphosphatase, could not be measured because arsenic trioxide interfered very strongly with the reaction involved in the assay of the phosphate group that is released by the action of the enzyme.

**Table 2:** Effects of 50 µM arsenic trioxide on the activities of phosphoenolpyruvate carboxykinase and pyruvate carboxylase. The experimental procedures are described in the Materials and Methods section. The *p* values refer to Student’s *t* test

Enzyme	Control	Arsenic trioxide (50 µM)	*p*
	nmol min^−^^1^ (mg protein)^−^^1^		
Phosphoenolpyruvate carboxykinase (PEPCK) (*n* = 4)	85.52 ± 2.67	48.66 ± 3.07	< 0.001
Pyruvate carboxylase (PC) (*n* = 3)	58.09 ± 2.97	0.22 ± 0.06	< 0.001

## Discussion

**Fig. 9:**
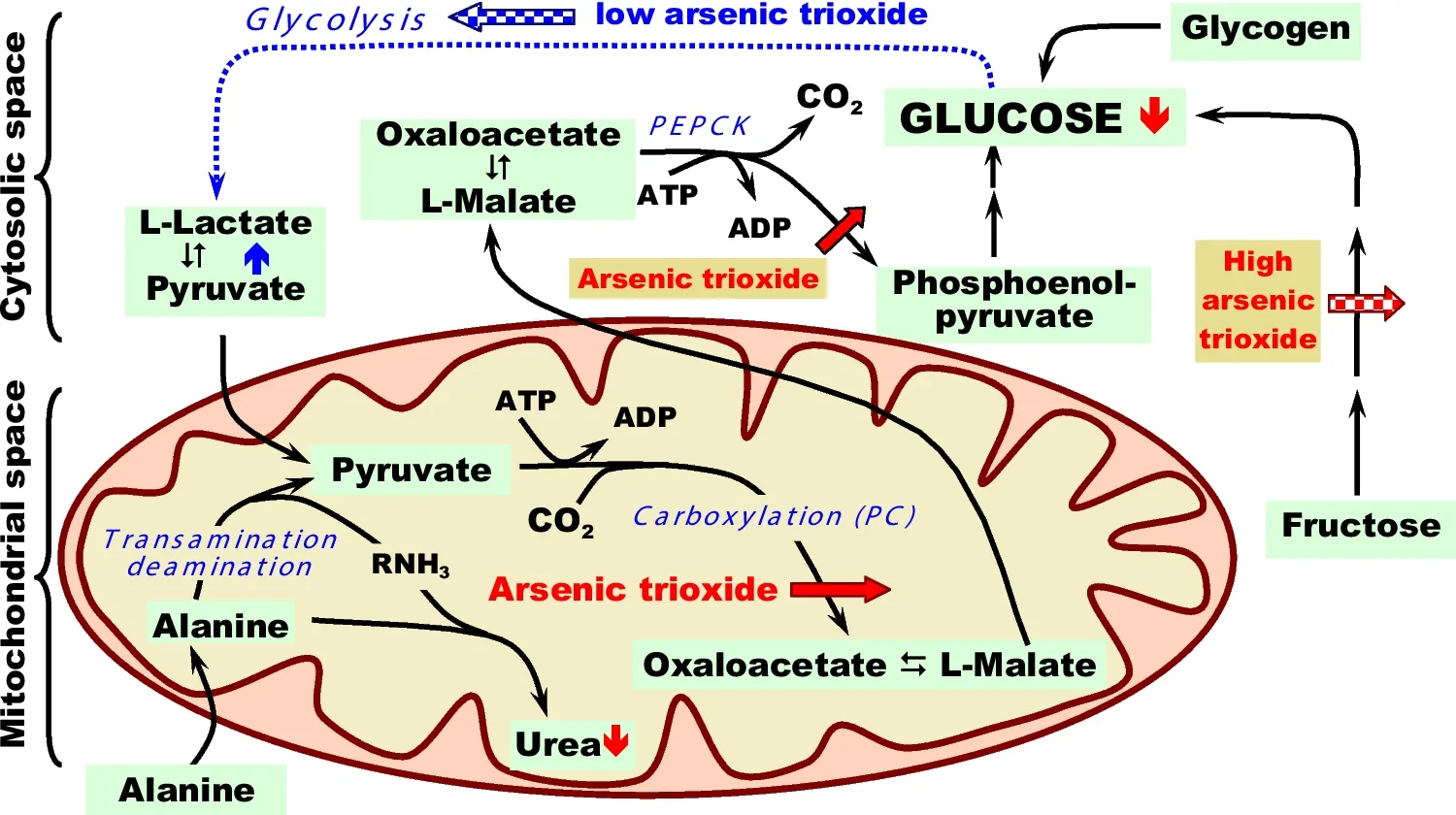
Scheme detailing in pictorial form the meaning of the major findings of the present work. Blue arrows denote stimulation and red arrows inhibition. Equilibrium reactions are represented by ↓↑. Intermediate steps leading to final products are represented as → →

The results of the present study indicate that arsenic trioxide modifies several metabolic fluxes in the liver in a reversible manner. The major findings are illustrated by Fig. 9. The compound strongly inhibited the gluconeogenic pathway initiating with three-carbon units, via the central precursor pyruvate. It inhibited carbon as well as nitrogen fluxes derived from the metabolism of alanine and stimulated glycolysis derived from glucosyl units generated by glycogen catabolism. It also inhibited fructose anabolism (glucose production) and increased catabolism (lactate and pyruvate production). Inhibition of fructose transformation into glucose, however, occurred at higher concentrations than the synthesis of glucose from lactate and alanine and was clearly incomplete even at the high concentration of 500 µM suggesting different action mechanisms. A characteristic of all metabolic effects of arsenic trioxide is the absence of correlation between oxygen uptake inhibition and gluconeogenesis inhibition. For example, under conditions in which lactate gluconeogenesis was operating, oxygen uptake was inhibited at concentrations that are higher than those already inhibiting gluconeogenesis. In the presence of alanine, no significant inhibition of oxygen uptake was found in spite of the strong inhibition of gluconeogenesis. In isolated mitochondria indeed, no significant effects on respiration were found for concentrations of up to 100 µM. Higher concentrations may eventually be inhibitory, but these and other observations suggest that inhibition of mitochondrial energy metabolism is not a prominent event among the actions of arsenic trioxide present in concentrations of up to 100 µM. These observations suggest that oxygen uptake inhibition in the liver, when it occurred under specific experimental conditions, was caused by conditions that are extraneous to the mitochondria itself. For example, inhibition of oxygen uptake in the presence of lactate could well be caused by the diminished need in ATP in consequence of the decreased rate of gluconeogenesis, which in turn was caused by inhibition of gluconeogenic key enzymes. This conclusion is indeed corroborated by the observation that, under these specific conditions, the arsenical is unable to cause a pronounced decline in the ATP content in the hepatic tissue. In this respect, arsenic trioxide differs markedly from sodium meta-arsenite, which consistently inhibits oxygen uptake by the liver in parallel with gluconeogenesis under the same conditions that were investigated in the present work (Melo et al. 2024).

As reemphasized by Fig. 9, the main mechanism by which arsenic trioxide inhibited gluconeogenesis from precursors involving pyruvate carboxylation was more likely the inhibition of at least two enzymes of the gluconeogenic pathway. These enzymes are pyruvate carboxylase (PC) and phosphoenolpyruvate carboxykinase (PEPCK). A likely mechanism of these inhibitions could be the interactions of arsenic trioxide with thiol groups. Arsenic trioxide and arsenicals in general are well known for their actions on thiol groups of cysteine residues, an action that has been appointed as being the main cause for the inhibition of a considerable number of enzymes (Knowles and Benson 1983; Thomas et al. 2001; Park and Butcher 2010). And, in the case of pyruvate carboxylase and phosphoenolpyruvate carboxykinase in particular, it has been amply demonstrated that in both inactivation of their thiol groups near the active center considerably diminishes their catalytic efficiency (Lewist et al. 1993; Palacián and Neet 1972). Among these two enzymes, inhibition of pyruvate carboxylase is probably the most decisive event for two reasons: (a) inhibition of this enzyme by arsenic trioxide is considerably more pronounced than the inhibition of PEPCK; (b) the flux control coefficient of PC largely exceeds that of PEPCK. In fact, the flux control coefficient of the PC can be estimated to be equal to 66.9% for the lactate concentration used in the present study (2 mM), whereas the flux control coefficient of PEPCK is only 1.95% under the same conditions (Groen et al. 1986; Bonetti et al. 2025). Considering, thus, the flux control coefficient of the PEPCK, very high degrees of inhibition would in principle be required for this enzyme to exert a significant influence on the gluconeogenic flux unless the inhibitory agent by itself changes the flux control coefficient. Other enzymes may eventually be involved, however, especially the fructose 1,6-bisphosphatase, which could not be measured in the present work because arsenic trioxide interferes with the current technique used to quantify phosphate. However, if inhibition of the fructose 1,6-bisphosphatase was an important inhibitory target of arsenic trioxide, the latter should also inhibit gluconeogenesis from glycerol, a phenomenon that was not observed. The hypothesis raised herein that pyruvate carboxylase is the main site of gluconeogenesis inhibition by arsenic trioxide corroborates the previous observation of Melo et al. (2024) which proposed that the inhibition of gluconeogenesis by sodium arsenite is largely the consequence of an inhibition of pyruvate carboxylation. At the same time, this interpretation weakens the proposition of Szinicz and Forth (1988) based mainly on experiments with isolated renal tubules. These authors proposed that inhibition of gluconeogenesis is the consequence of a diminution in the cellular concentration of acetyl-CoA, which is a positive modulator of pyruvate carboxylase (Jitrapakdee et al. 2008). Although this mechanism might also be contributing to the whole inhibitory effect, the strong direct action of arsenic trioxide on the activity of PC seems to be a more likely mechanism, especially because inhibition of gluconeogenesis develops rapidly even when there is no inhibition of oxygen uptake (with alanine gluconeogenesis, for example). The latter is an expected phenomenon if the availability of acetyl-CoA is substantially diminished.

Besides gluconeogenesis inhibition, several other modifications caused by arsenic trioxide can also be interpreted as a direct consequence of the actions on pyruvate carboxylase. For example, the increased pyruvate and lactate release caused by arsenic trioxide in the presence of alanine was possibly reflecting the accumulation of pyruvate whose transformation into oxaloacetate was inhibited. Lactate production also increased under such circumstances because of the lactate dehydrogenase equilibrium. The absence of effects on glycerol metabolism, on the other hand, is compatible with an inhibitory action on pyruvate carboxylase and phosphoenolpyruvate carboxykinase because the metabolic transformation of the former substrate does not require these two enzymes. Other inhibitory or stimulatory phenomena, however, are hard to explain based on factual evidence. This is clearly the case of glycolysis stimulation at low concentrations, the inhibition of glucose formation from fructose, and the increased rates of fructolysis. Fructose transformation into glucose is also independent of the activities of pyruvate carboxylase and phosphoenolpyruvate carboxykinase, but the pathway is also inhibited by arsenic trioxide. This suggests other inhibitory sites for the arsenical whose identification depends on further investigations. Inhibition of fructose transformation into glucose is actually a phenomenon that differed from lactate and alanine gluconeogenesis in that it was clearly incomplete, i.e., total inhibition was not achieved even at the very high concentration of 500 µM. The simultaneously slightly increased rates of lactate + pyruvate production (fructolysis) could be resulting from a deviation of phosphorylated fructose intermediates, which would otherwise be transformed into glucose.

The stimulatory action of arsenic trioxide on glycolysis occurs only at low concentrations. Maximal stimulation was found at a concentration close to the IC_50_ for the inhibition of lactate or alanine gluconeogenesis. The stimulatory effect vanished at higher concentrations, but it is difficult to ascertain if this occurred in consequence of an inhibition that superimposed on stimulation or if it is the consequence of some type of counter-regulation. Stimulation of glycolysis occurs when mitochondrial respiration is inhibited as a compensatory phenomenon for the diminished ATP synthesis in the mitochondria which leads to a diminished cellular energy charge (da Silva Simões et al. 2017; Pereira-Maróstica et al. 2023). However, in the present case, the stimulatory effect on glycolysis was not synchronous with oxygen uptake inhibition, which occurred only at higher concentrations. The data obtained herein, thus, do not allow for inference about the mechanism behind this effect. It should be stressed, however, that there are reports of arsenicals, such as sodium arsenate, that are able to increase glycolysis in the liver as well as in erythrocytes (Melo et al. 2024). An inhibition superimposing on stimulation at higher concentrations, on the other hand, is supported by previous observations that a great number of proteins are affected by arsenicals. Actually, a total of 200 enzymes have been reported to be inactivated by arsenicals in general (Shen et al. 2013; Zhang et al. 2015). The list includes not only enzymes catalyzing intermediate reactions in metabolism, including glycolysis, but also receptors and regulatory enzymes. How exactly this very complex net of effects manifested on the metabolic fluxes measured in the present work is very hard to discern with exactitude.

The results obtained herein indicate that several short-term effects of arsenic trioxide on liver metabolism are similar to those observed for sodium arsenite and also that they occur within a similar concentration range. Lactate gluconeogenesis inhibition by sodium arsenite, for example, occurs with a IC_50_ of 25 µM (Melo et al. 2024); the value is relatively close to the IC_50_’s for lactate and alanine gluconeogenesis inhibition caused by arsenic trioxide, which were 21.7 and 21.4 µM, respectively. With respect to glycolysis stimulation, arsenic trioxide was already active at the low concentration of 10 µM, a concentration at which sodium arsenite caused similar alterations (Melo et al. 2024). These concentrations are within the same range at which oxidative stress caused by arsenic trioxide has been observed in mammalian cellular systems (Alarifi et al. 2013; Kumar et al. 2014; Park et al. 2018). Oxidative stress has been regarded as one of the main causes of the toxical actions of arsenic trioxide in various tissues, including the liver (Mathews et al. 2006; Wen et al. 2025). In human hepatocellular carcinoma cells, it was found, for example, that arsenic trioxide increases the cellular levels of reactive oxygen species by 63.2% after 1 or 2 h at the concentration of 25.3 µM (Alarifi et al. 2013). At the same concentration and time, arsenic trioxide reduces the GSH levels by 44% and nearly doubles lipid peroxidation in the same cells. Taking, thus, into account the similar concentrations of arsenic trioxide required for both the metabolism disrupting effects described in the present work and the well-established oxidative stress induced by the compound, it seems reasonable to assume that similar concentrations will also be required to generate the corresponding toxic consequences in whole organisms. In cases of intentional or accidental poisoning, it looks highly probable that such concentrations are easily achieved if one considers the frequently observed fatal consequences. It should be emphasized here that there is an important difference between the toxicity in consequence of metabolism inhibition (this work) and toxicity originated from exacerbated oxidative stress. The latter usually arises from a very small fraction of the mitochondrial metabolism (rarely exceeding 2%; Murphy 2009) and its cumulative consequences manifest only after a relatively long time. Compared to the fast development of gluconeogenesis inhibition (not much more than 10 min), for example, the development of strong oxidative stress conditions requires much more time (hours) and can, thus, be considered a long-term or at least a medium-term effect (Alarifi et al. 2013; Kumar et al. 2014; Park et al. 2018). This fact also makes it unlikely that exacerbated oxidative stress could have contributed significantly to the metabolic effects found in the present work.

Reports of fatal outcomes when arsenic trioxide, either as trisenox or phenasen, are used in promyelocytic leukemia therapy are relatively rare. However, reports of relatively mild toxicity episodes are not uncommon (Au and Kwong 2008; Australian Government 2014; Liu et al. 2021). That the use of arsenic trioxide for cancer therapy is relatively safe is certainly linked to the plasma concentration that is effectively reached in the clinical procedures. Reports about the maximal systemic plasma concentration of arsenic trioxide in patients present variations depending on the administered dose and other factors including the assay system utilized. For the approximate dose of 10 mg, values between 1 and 7 µM have been reported with a tendency of lower values in more recent times (Shen et al. 1997; Jianhua et al. 1998; Au and Kwong 2008). Comparison of these concentrations with the concentrations that produce strong oxidative stress or pronounced disruption of liver metabolism explains quite well the practical observation that the clinical treatment with arsenic trioxide is relatively safe. Even so, the therapeutic and toxic concentrations are relatively close and the risk of accidental overdosing should not be neglected. For the liver, this is an issue that deserves attention when the compound is administered orally (Torka et al. 2015). In this case, the compound reaches the liver via the portal vein and not through the systemic circulation. Normally, the concentration of chemicals administered intragastrically in the portal vein greatly exceeds that in the systemic circulation (Matsuda et al. 2015) a situation in which the liver is exposed to an enhanced burden at least during a certain period of time.

In conclusion, the acute effects of arsenic trioxide described in the present work are likely to make a significant contribution to the general toxicity of the compound, especially when combined with the reported long-term induction of exacerbated reactive oxygen species (ROS) production which leads to the activation of apoptotic pathways among other deleterious consequences (Ling et al. 2017). When using the compound as a therapeutic agent, extreme care must be taken to avoid even mild overdosing as harmful and therapeutic levels seem to be relatively close to each other.

## Data Availability

Data are available on request from the corresponding author (Adelar Bracht, abracht@uem.br).
